# EMBO Molecular Medicine: Fast Forward

**DOI:** 10.1002/emmm.201303745

**Published:** 2014-01-09

**Authors:** Stefanie Dimmeler, Céline Carret, Roberto Buccione

## Abstract

These are exciting times for translational medicine as the convergence between fundamental and clinical research comes of age. The new EMBO Press publishing platform reinforces the standing of EMBO Molecular Medicine as the journal that matches high quality, novel research with rigorous editorial and ethical standards. It will also cement the journal's global reach and relevance - whether in highly active fields or explorative forays into emerging areas.

The ever shifting scientific publishing landscape poses new challenges and requires the freedom and flexibility to continuously adapt and evolve. EMBO is therefore launching its own publishing platform, EMBO Press, in collaboration with Wiley and HighWire Press. EMBO Press is uniquely positioned to build on the reputation our journals have built by publishing great science, and turn this to the advantage of the global life sciences community. As a prestigious independent publisher, free from ‘for-profit’ commercial objectives, EMBO Press can and will ensure that the interests of science are put at the heart of the publishing process – while promoting and applying, in our best tradition, high ethical and editorial standards and innovation to enhance the communication of research. All the income benefits from publishing will continue to support EMBO – and, as a consequence, the scientific community – through its many activities and initiatives.

*EMBO Molecular Medicine* has been published by Wiley since 2009. Wiley will now support editorial production and marketing of all four EMBO scientific journals. HighWire Press is at the forefront of digital scholarly publishing and will provide a consistent, reliable and innovative online platform for the journals. Many leading journals in the life sciences rely on HighWire Press and we look forward to cooperating with these journals on technology and policy developments. *EMBO Molecular Medicine* will be an integral part of this venture in applying and further developing the EMBO Press editorial policies — including transparent peer-review, scooping protection, referee cross-commenting and the source data initiative.

As a shared identity with the other three EMBO journals — *EMBO reports*, *The EMBO Journal* and *Molecular Systems Biology*, EMBO Press will serve as a platform for our joint policies and initiatives, guided by our international advisory boards of leading researchers and the scientific community. A single, strong voice pushing for greater fairness, transparency and innovation in the interests of life scientists will contribute to improving the way that science is communicated and used and researchers are assessed.

What does this mean in particular for *EMBO Molecular Medicine*? *EMBO Molecular Medicine* will continue to be a highly selective open access journal dedicated to research at the interface between clinical research and basic science. The most obvious and immediate change is the launch of the new journal website, alongside the EMBO Press website. Over the next few months, we will make significant enhancements to help scientists present their data, making it more accessible and reusable, while improving the experience for both authors and readers. We are clearly moving forward into an exceptional time for translational medicine as the convergence between fundamental and clinical research comes of age. EMBO press will help establish *EMBO Molecular Medicine* as a medium for high quality research whether in highly active areas or new, explorative forays into emerging research areas.

We hope that our authors and readers will agree and support us on this journey and help move us forward.

Find out more at embopress.org. As always, we welcome your comments as they will inform future enhancements.

## Conflict of interest

The authors declare that they have no conflict of interest.

